# Elevational Variation in Soil Amino Acid and Inorganic Nitrogen Concentrations in Taibai Mountain, China

**DOI:** 10.1371/journal.pone.0157979

**Published:** 2016-06-23

**Authors:** Xiaochuang Cao, Qingxu Ma, Chu Zhong, Xin Yang, Lianfeng Zhu, Junhua Zhang, Qianyu Jin, Lianghuan Wu

**Affiliations:** 1 State Key Laboratory of Rice Biology, China National Rice Research Institute, Hangzhou, 310006 China; 2 Ministry of Education Key Laboratory of Environmental Remediation and Ecosystem Health, College of Environmental and Resource Sciences, Zhejiang University, Hangzhou, 310058 China; USDA-ARS, UNITED STATES

## Abstract

Amino acids are important sources of soil organic nitrogen (N), which is essential for plant nutrition, but detailed information about which amino acids predominant and whether amino acid composition varies with elevation is lacking. In this study, we hypothesized that the concentrations of amino acids in soil would increase and their composition would vary along the elevational gradient of Taibai Mountain, as plant-derived organic matter accumulated and N mineralization and microbial immobilization of amino acids slowed with reduced soil temperature. Results showed that the concentrations of soil extractable total N, extractable organic N and amino acids significantly increased with elevation due to the accumulation of soil organic matter and the greater N content. Soil extractable organic N concentration was significantly greater than that of the extractable inorganic N (NO_3_^−^-N + NH_4_^+^-N). On average, soil adsorbed amino acid concentration was approximately 5-fold greater than that of the free amino acids, which indicates that adsorbed amino acids extracted with the strong salt solution likely represent a potential source for the replenishment of free amino acids. We found no appreciable evidence to suggest that amino acids with simple molecular structure were dominant at low elevations, whereas amino acids with high molecular weight and complex aromatic structure dominated the high elevations. Across the elevational gradient, the amino acid pool was dominated by alanine, aspartic acid, glycine, glutamic acid, histidine, serine and threonine. These seven amino acids accounted for approximately 68.9% of the total hydrolyzable amino acid pool. The proportions of isoleucine, tyrosine and methionine varied with elevation, while soil major amino acid composition (including alanine, arginine, aspartic acid, glycine, histidine, leucine, phenylalanine, serine, threonine and valine) did not vary appreciably with elevation (*p*>0.10). The compositional similarity of many amino acids across the elevational gradient suggests that soil amino acids likely originate from a common source or through similar biochemical processes.

## Introduction

Paradigms on terrestrial nitrogen (N) cycling posit that soil organic N must be converted into inorganic N (e.g., NO_3_^-^, NH_4_^+^) by microorganisms prior to becoming available to plant roots. Thus, N mineralization has been viewed as the bottleneck in plant N nutrition [1], but the role of soil dissolved organic N in meeting the nutritional requirements of forest and agricultural plants has increased since the 1990s [2–5]. Numerous laboratory and field studies have demonstrated that both mycorrhizal and non-mycorrhizal plants can directly absorb soil organic N, especially amino acids, thereby circumventing the traditional mineralization bottleneck [6–8]. In some low N-input and cold ecosystems, pools of amino acid N in soils have been shown to rival or exceed those of mineral N. So plant uptake of amino acids has the potential to be a primary factor in ecosystem function and vegetation succession [9–12].

The ability of plants to acquire N in both inorganic and organic forms suggests that the concentration and proportion of these forms in soil are important determinants of plant N nutrition and species diversity. Whereas soil biogeochemists have a well-developed understanding of the controls over the production, consumption and transformation of mineral forms of N in soil, and how they affect the availability of N for plant uptake, we are just beginning to understand the behavior of amino acids in soil. Clearly, the question regarding the importance of amino acids in ecosystem N cycling requires knowledge of the concentrations of soil amino acids. It is well established that free amino acids in soil typically account for less than 1% of the pool of dissolved organic N, and their concentrations in soil solution are on the order of 0.1–50 μmol L^-1^ [13–15]. Further studies have demonstrated that soil amino acid composition is dominated by a small number (generally 6) of abundant amino acids including: alanine, asparagine, aspartic acid, glutamine, glutamic acid and histidine [16, 17]. However, these studies were limited to the free amino acids in soil solution or dissolved organic N pool and neglected the fact that as much as 88–92% of amino acids can be readily adsorbed to the soil solid phase [18]. In addition, approximately 40% of soil N is present in the form of polymers, such as proteins and peptides [19], and 30–45% of these polymers are present as amino acids after proteolysis [20]. Therefore, studies should include a measurement of the total concentration of amino acids to obtain a direct comparison of the different N forms, which is critical for developing hypotheses and understanding soil N dynamics.

Generally, 20 common amino acids exist in soil, and plants have the capacity to take up a variety of them [7, 21, 22]. Several intrinsic properties of amino acids that affect their behavior in soil have been proposed, including molecular weight [23–25], charge characteristics [26, 27] and N content [28, 29]. Rothstein demonstrated that the processes of mineralization, microbial assimilation and adsorption to soil solids significantly influenced the concentration of amino acids and their contribution to plant nutrition [30]. In addition, changes in climate significantly influence plant productivity and the characteristics of soil, such as litter input, seasonal freeze-thaw and dry-rewet events, root secretion and microbial activity [31–33], which also greatly affect soil amino acid composition. Temporal variation in concentration of individual amino acids has been reported in arctic terrain [34] and temperate grasslands [16], and elevation is often used to study the effects of climatic variables on the basic dynamics of soil properties [35]. However, fewer studies have investigated the spatial variation in amino acid behavior along the elevational gradient.

Here we characterized the composition of soil amino acids and inorganic N along an elevational gradient at Taibai Mountain, China. A previous study demonstrated that temperature decreased and precipitation increased with elevation on Taibai Mountain [36]. Across the elevation gradient, several soil types are present that developed in response to the climate, parent material, topography, vegetation, time and human activities. Therefore, soils were sampled at 13 different elevations between 470 m and 3760 m at approximately 250–300 m intervals along the elevational gradient of Taibai Mountain. We hypothesized that the concentrations of amino acids would increase along the elevational gradient as plant-derived organic matter accumulated and N mineralization and microbial immobilization of amino acids slowed with reduced soil temperature and a higher proportion of recalcitrant N. Second, we hypothesized that the composition of individual hydrolyzable amino acids in soil would vary along the elevational gradient. Specifically, we predicted amino acids with simple molecular structure would be prevalent at low elevations due to rapid plant growth and larger inputs of labile C and N. Furthermore, amino acids with complex aromatic structure and high molecular weight would be prevalent at high elevations resulting from the lower temperature and smaller inputs of labile C and N.

## Materials and Methods

### Study site and soil collection

The study area was conducted at Taibai Mountain (107°19′-107°58′E and 33°45′-34°10′N), which is the highest mountain in the Qinling Range of eastern mainland China. The Qinling Range varies in elevation from 470 m to 3760 m above sea level, and it is the climate demarcation line between the northern and southern regions of China as well as the watershed between the Yellow and Yangtze Rivers. Because of its particular location and significant elevation, Taibai Mountain exhibits a high degree of variation in climate, vegetation form and soil type. The northern slope of Taibai Mountain falls into five climate zones: warm temperate, temperate, cold temperate, cold, and alpine cold. The average annual temperature varies from 11.0°C (1250 m) to 1.1°C (3250 m) [35], and the mean annual precipitation is 751.8 mm, which is primarily concentrated between July and September, a period that accounts for 50% of the total precipitation. These diverse environmental factors have helped create a variety of soil types along the elevational gradient, including Lou soil (Earth-cumulic Orthic Anthrosols, <750 m), Cinnamon soil (Typic Hapli-Ustic Argosols, 750–1400 m), Brown soil (TypicHapli-Udic Argosols, 1400–2400 m), Dark brown soil (Aci-Udic Cambisols, 2400–3300 m), Subalpine forest-meadow soil (Mollic Gryic Cambisols, 3300–3500) and Alpine meadow soil (Umbric Cryic Cambisols, >3500 m) [37].

We sampled soil at 13 elevations at approximately 250–300 m intervals (fluctuating up or down by 20 m at any given elevation) on the northern slope of Taibai Mountain at elevations of 470 m, 750 m, 1030 m, 1310 m, 1590 m, 1870 m, 2150 m, 2430 m, 2710 m, 2990 m, 3270 m, 3550 m, and 3760 m. Soil samples were collected from three replicate plots (approximately 4 m×3 m) per elevation. Plots were separated by 2 m along the elevation contour, and the replicated plots at each elevation have similar elevations, vegetative communities and soil textures. For each plot, we collected 12 soil cores (subsamples per plot) sampling followed an S-shaped sampling pattern within each plot, and subsamples were thoroughly mixed. Soil samples were collected after the snow had completely melted in August 2012. Individual cores were separated into organic and mineral horizons per plot. Horizon subsamples were mixed to create composite organic and mineral horizon samples per plot. The buried organic horizons that less than 0.5 cm thick were combined with the mineral soil.

Upon collection, soil samples were immediately hand-sorted to remove live plant material and other woody debris. They were then mixed by hand for several minutes and transported to the laboratory on an ice bag within 48 hours of collection. Our soil sampling activities were permitted by the Administration of Taibai Mountain Natural Reserve of Shaanxi Province. This field study did not involve any endangered or protected species.

### Extraction of soil samples

In the laboratory, soils were passed through a 2-mm sieve and air-dried. Three 10-g sub-samples of each soil sample were mixed with 0.5 mol L^-1^ K_2_SO_4_ at a soil:solution ratio of 1:5 (w:v), shaken (150 r, 60 min), centrifuged (4000 g, 10 min), and filtered through Whatman#42 filter paper [38]. We stored the extracts at -80°C before analysis. NO_3_^−^-N and NH_4_^+^-N were determined by segmented continuous flow analysis, and extractable total N was measured by determining NO_3_^−^-N with ultraviolet spectrophotometric measurement following alkaline potassium persulfate digestion [39]. Extractable organic N was calculated based on the difference in the concentrations of extractable total N and extractable inorganic N (NO_3_^−^-N+NH_4_^+^-N). Free amino acids in soils were extracted with deionized H_2_O at a soil:solution ratio of 1:5 (w:v) as described above. Concentrations of soil adsorbed amino acids were calculated as the total amino acids extracted with 0.5 mol L^-1^ K_2_SO_4_ minus the free amino acids [40]. Soil free amino acid and adsorbed amino acid concentrations were determined by the ninhydrin method [13], removing NH_4_^+^-N according to the method of Warren and Adams [41]. Although we divided the soil into its organic and mineral horizons within a soil core, the concentrations of extractable N in soils were calculated for the organic and mineral horizons combined because the organic horizon represented a progressively greater fraction of the total core volume across the different elevations, ranging from 0% at 470 m to 48% at 3270 m.

### Analysis of hydrolyzable amino acids

Protein-bound amino acids were liberated by acid hydrolysis of soil containing 10 mg N in 6 mol L^-1^ HCl (24 h, 105°C). After acid hydrolysis, hydrolysates were vacuum-filtered by syringe through Whatman 0.2 μm GD/X polyvinylidene fluoride membrane filters into 2.0-ml sterile cryovials, dried using a rotary evaporator (<45°C), and re-dissolved in 4 ml 0.05 mol L^-1^ HCl within the evaporation flasks. The final solution was purified (through desalting and removal of interfering organic compounds) using a polypropylene sample-preparation column filled with 3-g Dowex 50WX8 cation exchange resin (100±200 mesh, Bio-Rad, USA). The solution was neutralized by the addition of NaOH to obtain a pH of 6.5–6.8, collected in a conical flask and immediately concentrated to 2 ml by rotary evaporation. Single amino acids were detected using an amino acid analyzer (Hitachi, L8900, Japan), and we identified 17 of the 20 primary L-amino acids with the exceptions of tryptophan, asparagine and glutamine (asparagine and glutamine were converted into aspartic acid and glutamic acid, whereas tryptophan was damaged during the hydrolysis procedure). To test the recovery efficiency of our purification procedure, 1 ml internal standard solution containing 50 μg ml^-1^ of the 17 amino acids investigated was added to the column in 0.05 mol L^-1^ HCl. The average recovery efficiency of our purification procedures was 96±1%. Non-hydrolyzable N (N in the residue following acid hydrolysis) was calculated as the difference in concentration between soil total N and total hydrolyzable N.

## Statistical Analysis

All statistics, including analysis of variance (ANOVA) and least significant means, were performed using the SPSS system for Windows, version 14.0 (SPSS Inc., USA). Normality and homogeneity of variance were determined from Shapiro-Wilk's statistics and Levene's Test, respectively, and data were log_10_ transformed to meet the assumptions when required. Duncan’s least significant difference test was used to distinguish among individual mean values where applicable with a confidence level of *p*<0.05. Relationships between soil parameters and the different forms of N were tested by Pearson’s correlation analysis. In order to test whether soil hydrolyzable amino acid composition varies across the montane gradient, we first performed the principal component analysis (PCA) of the 17 different hydrolyzable amino acidcon centrations. Then the results from PCA analysis were used to perform the simple linear regressions and determine if variation in PCA scores was explained by elevation thereby indicating an elevational gradient in amino acid composition. Finally, we performed separate linear regressions for each of the 15 amino acid composition to determine whether each varied by elevation.

## Results

### Soil properties

Soil physical and chemical properties varied across the elevational gradient (Table 1). Soil organic matter, total N, and alkali-hydrolyzable N content generally increased with elevation and reached their highest values at 3550 m. In contrast, soil pH decreased progressively along the elevational gradient, varying from 7.79 (470 m) to 5.25 (3760 m). Soil C/N ratio, concentrations of soil available phosphorus, available potassium, exchangeable calcium and exchangeable magnesium did not exhibit any consistent variation along the elevational gradient.

**Table 1:** Basic soil properties along the elevational gradient of Taibai Mountain. Values represent the mean ± SE (standard error), n = 3.

Elevation	pH	OM ^†^	TN	C/N	Avail-N	Avail-P	Avail-K	Exchange- Mg	Exchange- Ca
m		g kg^-1^	g kg^-1^	/	mg kg^-1^	mg kg^-1^	mg kg^-1^	mg kg^-1^	mg kg^-1^
470	7.79±0.2a^¶^	17.7±0.7i	1.14±0.1f	9.02±0.5e	50.5±3.7h	11.8±0.7b	257±3.7a	172±5.9g	3986±155b
750	7.31±0.3a	42.9±1.8h	1.72±0.1f	14.5±1.2ab	67.0±1.8h	6.95±0.2ef	72±7.0c	74±1.5h	898±33h
1030	7.30±0.4a	53.9±1.2 gh	3.39±0.2e	9.22±0.7e	150±3.9g	9.57±1.2cd	273±5.2a	480±5.4a	3714±195bc
1310	6.85±0.1b	62.8±6.4efg	3.81±0.3de	9.56±0.8de	182±17fg	9.36±0.9cd	152±13b	262±5.6de	2789±76efg
1590	6.58±0.2b	59.1±6.3fgh	3.54±0.3de	9.70±0.5de	146±16g	12.1±0.3b	114±2.5bc	170±4.8g	2226±157fg
1870	6.25±0.6bc	75.3±5.0ef	3.76±0.2de	11.6±0.9cde	194±12ef	10.2±0.7bcd	107±17bc	191±11fg	2428±163fg
2150	6.48±0.5b	96.3±3.0cd	4.47±0.1cd	12.5±0.2bc	232±13de	10.7±0.4bc	107±5.1bc	228±12ef	2576±188efg
2430	6.01±0.4bc	107±4.0c	4.87±0.2bc	12.7±0.3abc	293±4.8c	10.4±0.5bcd	124±8.2b	366±12b	3806±176bc
2710	6.10±0.5bc	81.6±3.2de	4.04±0.2cde	11.7±0.2cd	247±8.5d	8.90±0.3cde	230±32a	321±6.3c	3353±118cd
2990	5.72±0.3c	142±11ab	5.69±0.6b	14.9±1.6a	342±13b	9.36±0.9cd	109±4.2bc	494±18a	4770±191a
3270	5.73±0.2c	110±8.2c	5.75±0.2b	11.1±0.5cde	318±21bc	6.15±0.2f	125±7.0b	276±14d	3228±209cde
3550	5.26±0.2d	158±10a	8.26±0.7a	11.1±0.6cde	493±15a	10.7±0.5bc	137±2.9b	285±15cd	2821±119def
3760	5.25±0.3d	131±8.1b	5.78±0.3b	13.1±0.6abc	306±17bc	16.2±0.2a	143±4.6b	221±11ef	2027±84g
Average	6.36	87.6	4.32	11.6	232	10.2	150	272	2971

^¶^ Values followed by different letters within the same column are significantly different (*P*<0.05) according to Duncan’s multiple range test.

^†^OM = organic matter, TN = total nitrogen, Avail-N = alkali-hydrolyzable N.

### Concentrations of soil amino acids and inorganic N

**Fig 1:**
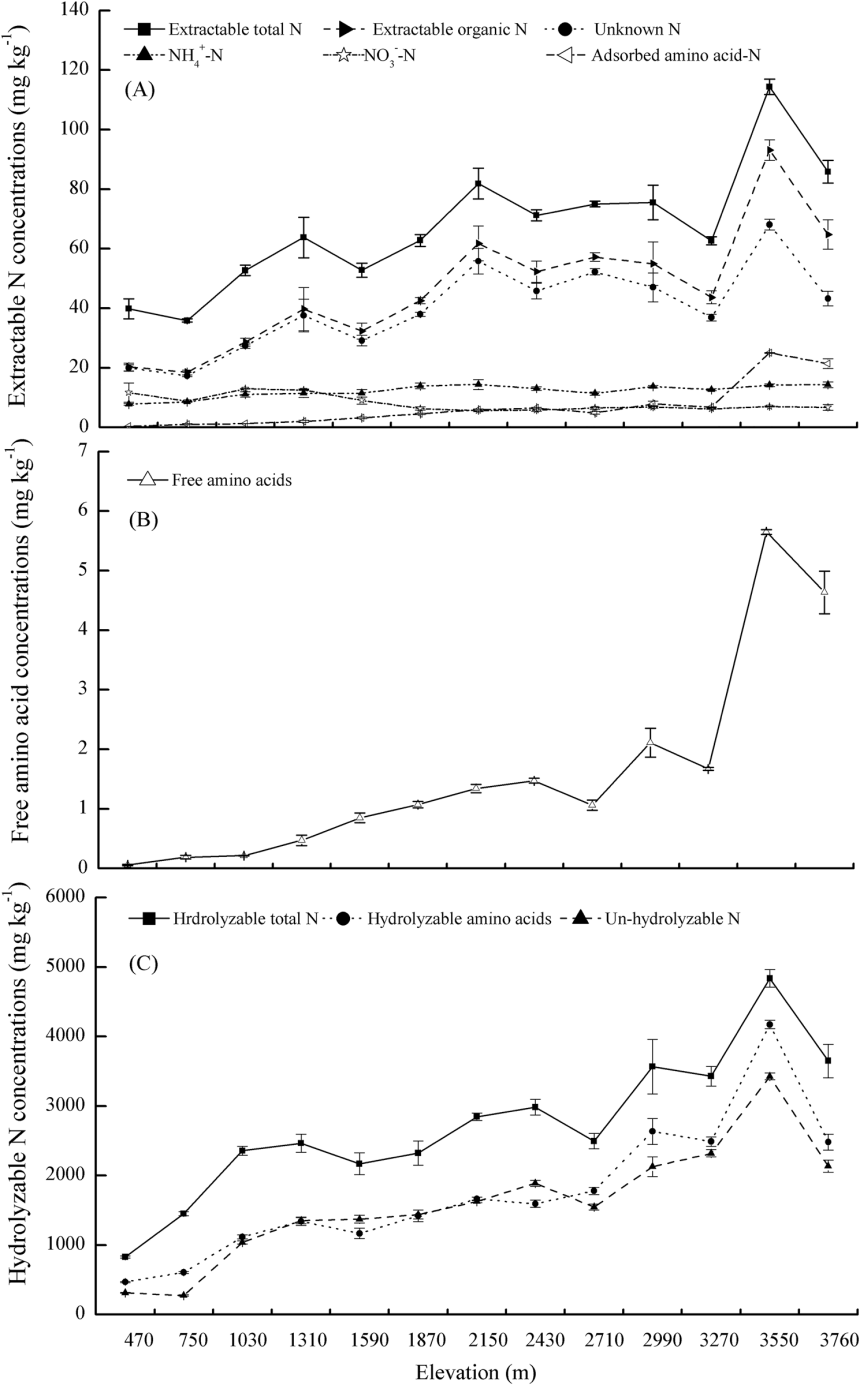
Concentrations of the different extractable N forms (A), free amino acids (B) and different hydrolyzable N forms (C) in the thirteen sampled elevations. Error bars represent standard error (n = 3).

Concentrations of extractable total N, extractable organic N, and adsorbed amino acids in soils increased with elevation (Fig 1A), and their extractable organic N concentrations were significantly greater than the concentrations of extractable inorganic N (including NO_3_^−^-N and NH_4_^+^-N). NO_3_^−^-N concentrations exhibited two distinct signals, high concentrations from 470–1600 m and significantly reduced concentrations from 1600–3700 m, but there were no significant differences in concentrations at the elevations from 1600–3760 m. In addition, the proportions of soil extracted NH_4_^+^-N and unknown N varied slightly along the elevational gradient, averaging 18.9% and 58.3% of the extractable total N concentration. Soil free amino acids gradually increased from 0.06 mg kg^-1^ to 5.6 mg kg^-1^ along the elevational gradient, and their mean concentration was 1.1 mg kg^-1^, representing 20% of the soil adsorbed amino acid concentration (approximately 5.5 mg kg^-1^, Fig 1A and 1B). Similarly, concentrations of hydrolyzable amino acids and hydrolyzable total N all increased significantly by an order of magnitude along the elevational gradient, and ranged from 466 and 825.6 mg kg^-1^ at 470 m to 4174 and 4836.7 mg kg^-1^ at 3550 m (Fig 1C).

### Composition of soil hydrolyzable amino acids

**Fig 2:**
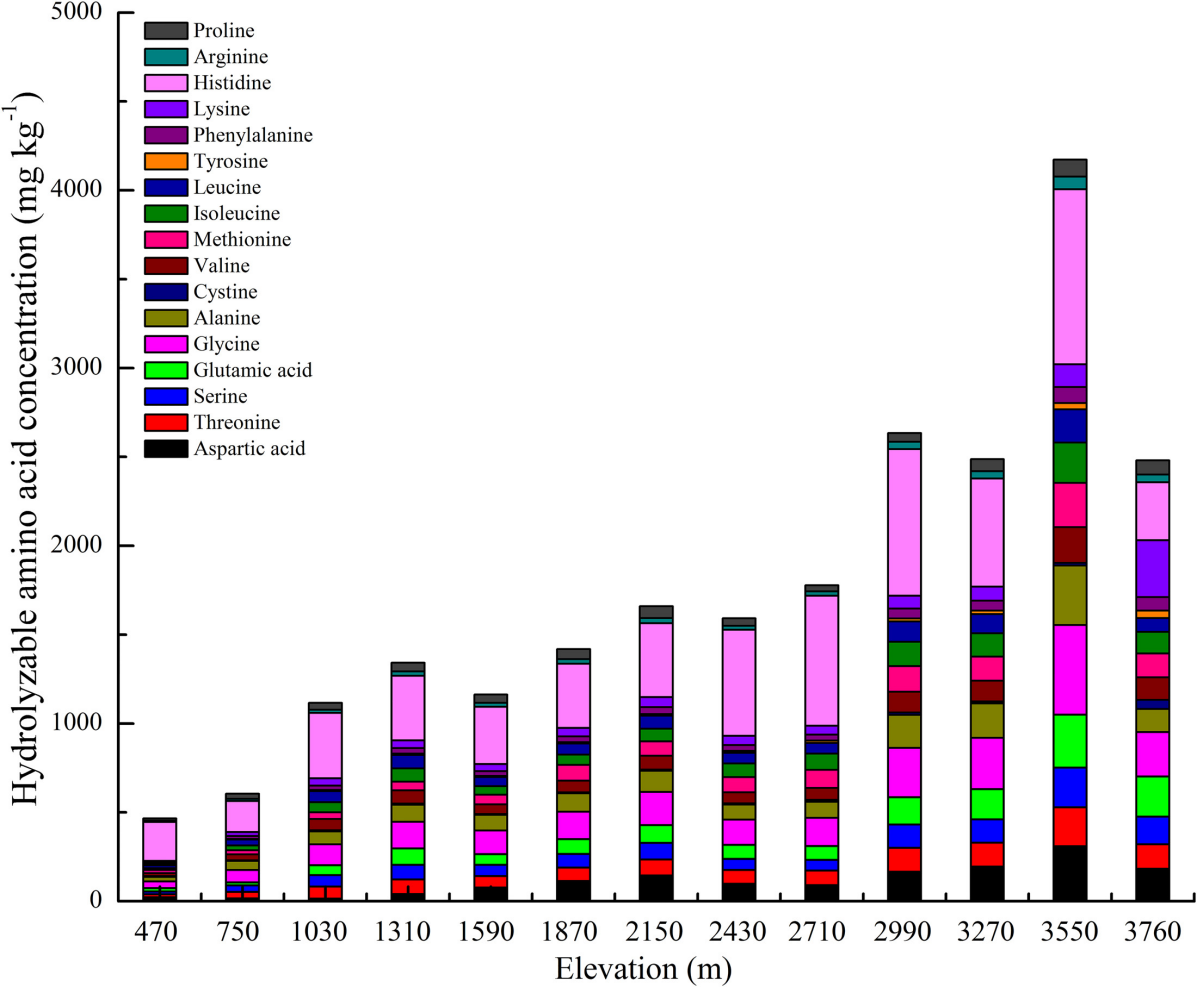
Concentrations of soil individual hydrolyzable amino acids in the thirteen sampled elevations. Error bars represent standard error (n = 3).

Soil individual amino acid concentration was diverse with seventeen of the twenty different amino acids detected after acid hydrolysis (Fig 2). Concentrations of the individual amino acids all increased significantly by an order of magnitude along the elevational gradient and reached their highest values at 3550 m. Across the elevational gradient, concentrations of the seven dominant amino acids ranged from 24.7–334.4 mg kg^-1^ for alanine, 14.2–308.4 mg kg^-1^ for aspartic acid, 38.0–505.0 mg kg^-1^ for glycine, 16.5–297.2 mg kg^-1^ for glutamic acid, 173.8–986.0 mg kg^-1^ for histidine, 15.7–223.1 mg kg^-1^ for serine and 16.6–220.4 mg kg^-1^ for threonine. On average, the seven major amino acids accounted for 69.6% of the concentration of soil total hydrolyzable amino acids. However, amino acids with high molecular weight and complex aromatic structure (such as tyrosine, proline, arginine and phenylalanine) constituted only a small proportion of the total amino acid composition at the different elevations.

**Fig 3:**
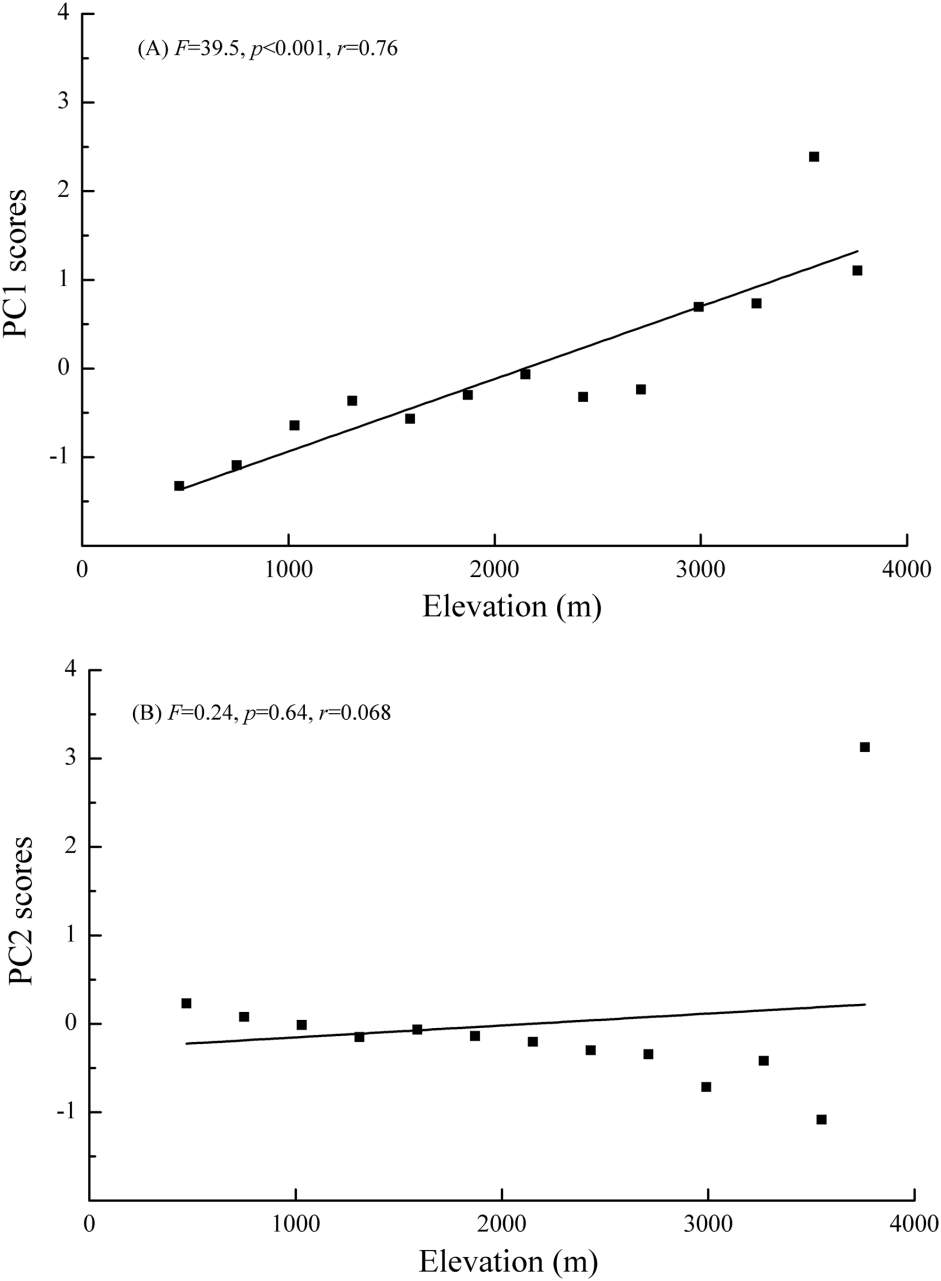
Elevational variations of the PC1 (A) and PC2 (B) score along a montane gradient. The best-fit lines, *F* values, goodness of fit (*r*) and *p*-values are provided.

**Fig 4:**
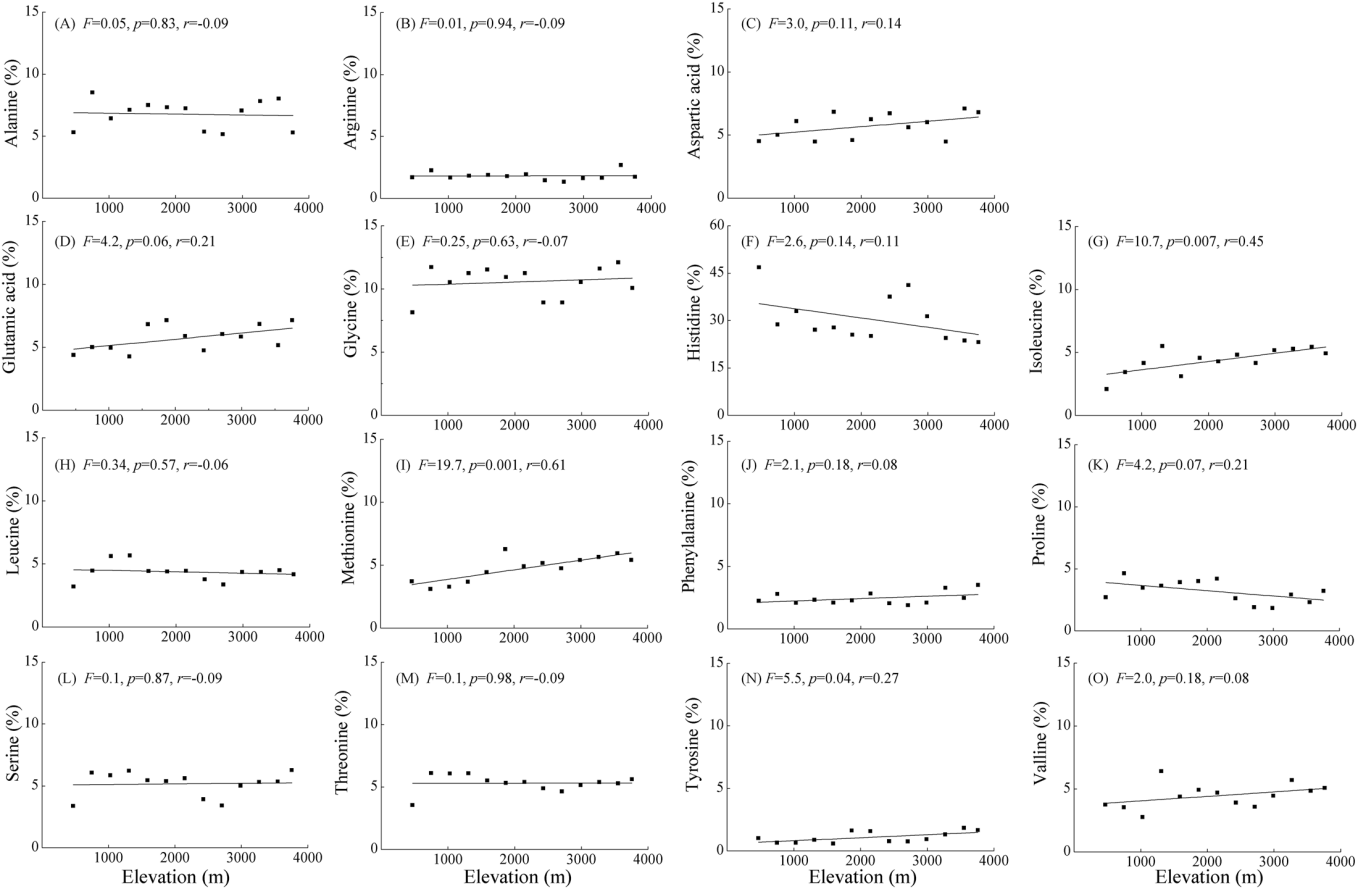
Simple linear regression of the relationships between the composition of individual amino acids contained in PC1 and elevation. The best-fit lines, *F* values, goodness of fit (*r*) and *p*-values are provided.

After the process of principal component analysis (PCA), we retained two principal components (PCs) with eigenvalues greater than unity and explaining 96.0% of the variance or information contained in the original data (Table 2). PC1 explained 84.3% of the variance and represents an aggregate index of most (15 of 17, Table 2) amino acids. PC2 explained 11.7% of the variance and represents an aggregate index of cystine and lysine. PC1 score was positively associated with the elevation (Fig 3A) thereby indicating that amino acid composition varied by elevation. Next, we performed 15 simple linear regression to determine whether any of this subset of amino acid composition was positively associated with elevation. The multiple simple linear regression analyses indicated that only three amino acid compositions {*e*.*g*. isoleucine (G), methionine (I), tyrosine (N)} increased along the elevational gradient (*p*<0.05). Two additional amino acids {*e*.*g*. glutamic acid (D), proline (K)} had a marginally significant (0.05<*p*≤0.10) positive association with elevation, and ten others {*e*.*g*. alanine (A), arginine (B), aspartic acid (C), glycine (E), histidine (F), leucine (H), phenylalanine (J), serine (L), threonine (M), and valine (O)} had not appreciable association with elevation (*p*>0.10) (Fig 4). In contrast, the relationship between PC2 and elevation was not significant (Fig 3B), and correspondingly, cystine and lysine composition showed no significant association with elevation (*p*>0.25).

**Table 2:** Loadings of the 17 different hydrolyzable amino acid variables on two significant principal components for the thirteen soils.

Variable	Communalities extraction	Principal components	
	(%)^†^	PC1^‡^	PC2
Aspartic acid	0.92	0.96	
Threonine	0.99	0.99	
Serine	0.98	0.99	
Glutamic acid	0.99	0.99	
Glycine	0.99	0.98	
Alanine	0.98	0.95	
Cystine	0.96		0.81
Valine	0.99	0.99	
Methionine	0.96	0.97	
Isoleucine	0.98	0.98	
Leucine	0.97	0.94	
Tyrosine	0.97	0.89	
Phenylalanine	0.99	0.99	
Lysine	0.99		0.76
Histidine	0.81	0.75	
Arginine	0.99	0.99	
Proline	0.82	0.90	
Eigenvalues		14.3	2.0
Variance explained		84.3%	11.7%

^†^The values in this column indicate the proportion of each variable's variance that can be explained by the principal components. Variables with high values are well represented in the common factor space, while variables with low values are not well represented.

^‡^Principal components analysis was based on the mean values of the 17 different amino acid composition. PC loadings <0.75 are not shown.

**Fig 5:**
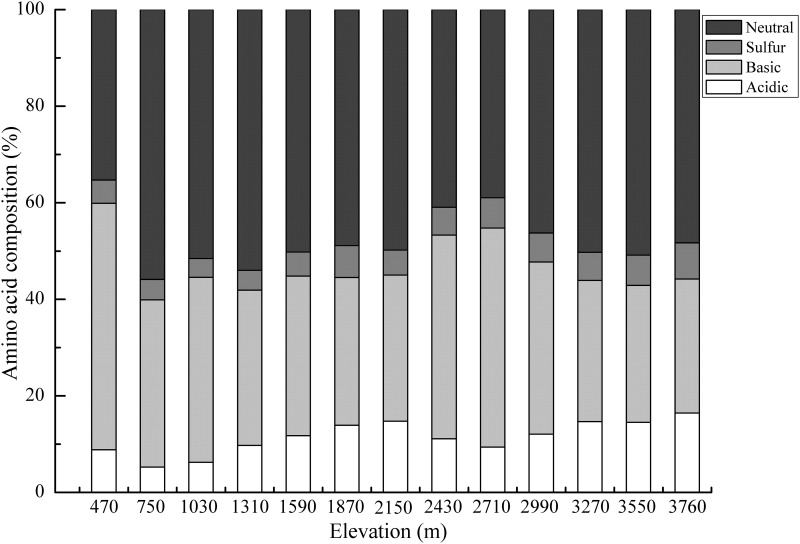
Proportions of the neutral, basic, acidic, and sulfur amino acids accounting for soil total hydrolyzable amino acids in the thirteen sampled elevations.

**Fig 6:**
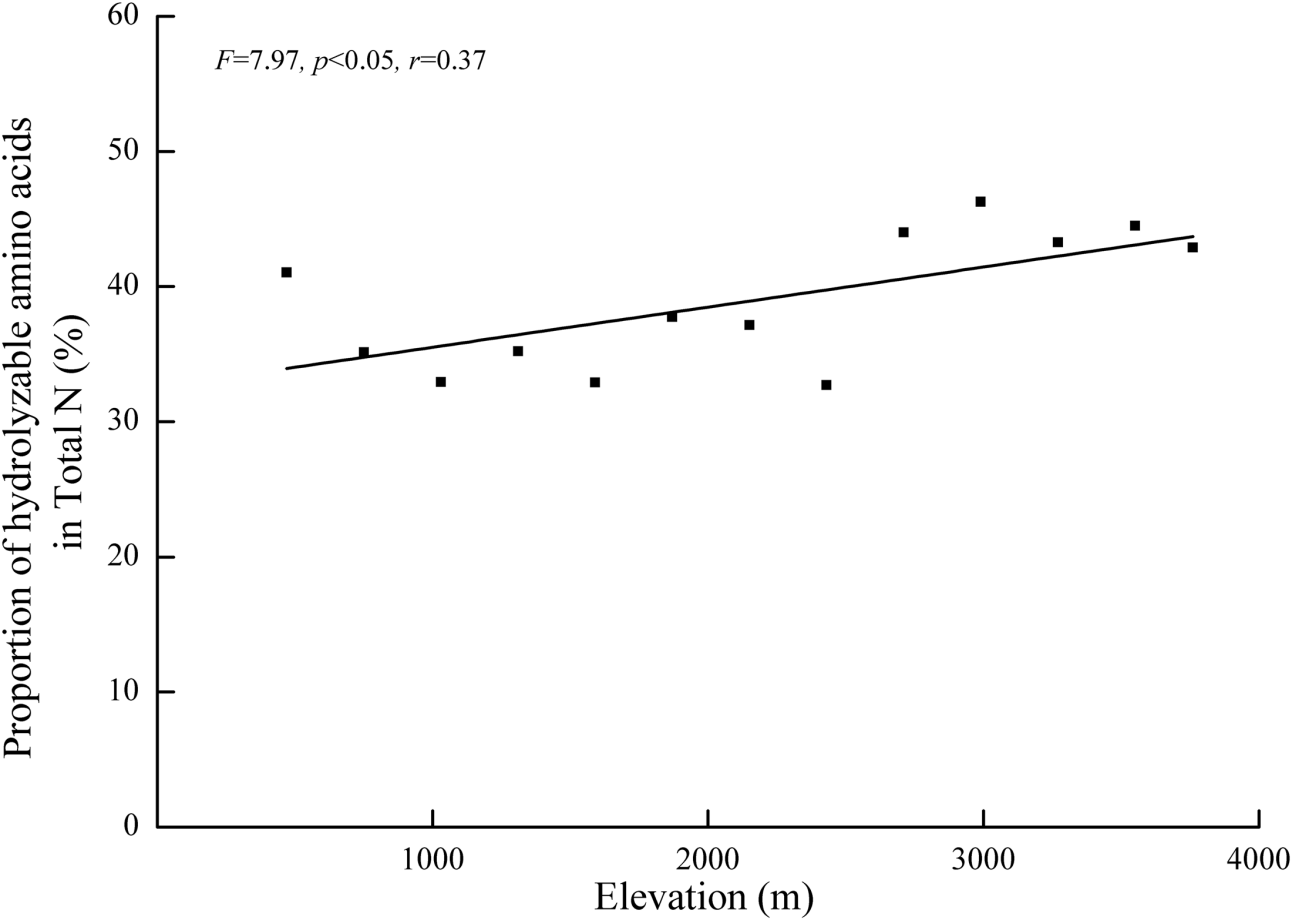
Elevational variations of the proportion of hydrolyzable amino acids that accounted soil total N content across the thirteen sampled elevations. The best-fit lines, *F* values, goodness of fit (*r*) and *p*-values are provided.

According to the functional classification, neutral amino acids accounted for 47.8% of the soil total hydrolyzable amino acids (Fig 5). The proportions of the other amino acids were as follows: basic amino acids (35.5%), acidic amino acids (11.4%) and sulfur amino acids (5.4%). Though soil hydrolyzable amino acid concentrations varied widely, its proportions of soil total N exhibited minimal variation across the thirteen soil sampled elevations (from 32.7% to 46.3%) (Fig 6, *p*<0.05, *r* = 0.65).

### Relationship among soil properties, inorganic N and amino acid N

Pearson correlation analysis demonstrated that basic soil properties (*e*.*g*. pH, organic matter, total N and alkali-hydrolyzable N) and different forms of soil N (*e*.*g*. extractable total N, NO_3_^−^-N, NH_4_^+^-N, extractable organic N, extractable unknown- N, adsorbed amino acids, free amino acids and hydrolyzable amino acids) were significantly related to elevation (Table 3, *p*<0.05). Specifically, soil extractable total N, NH_4_^+^-N, extractable organic N, adsorbed amino acids, free amino acids and hydrolyzable amino acids were positively related to soil organic matter, total N and alkali-hydrolyzable N contents (*r* = 0.57~0.91, *p*<0.05), but were negatively related to soil pH and NO_3_^−^-N content (*r* = -0.35~-0.86, *p*<0.05, except HAAs). In addition, there were significant correlations between soil adsorbed amino acids and free amino acids/hydrolyzable amino acids (*r* = 0.79~0.98, *p*<0.01).

**Table 3:** Pearson correlations among basic soil properties, inorganic N and amino acid N. Only significant correlations are presented.

	Elevation	ETN^†^	NO_3_^-^	NH_4_^+^	AAAs	UKN	EON	FAAs	HAAs	pH	OM	TN
ETN (mg kg^-1^)	0.83***											
NO_3_^-^ (mg kg^-1^)	-0.72**											
NH_4_^+^ (mg kg^-1^)	0.79**	0.64*	-0.41*									
AAAs (mg kg^-1^)	0.81***	0.77**	-0.35*	0.48*								
UKN (mg kg^-1^)	0.77*											
EON (mg kg^-1^)	0.75**	0.98***	-0.43*		0.79**	0.95*						
FAAs (mg kg^-1^)	0.83***	0.79***	-0.36*	0.49**	0.98***		0.78					
HAAs (mg kg^-1^)	0.88***	0.75*		0.47*	0.79**		0.64*	0.72*				
pH	-0.98**	-0.75*	0.52*	-0.59*	-0.79*		-0.78**	-0.81*	-0.86**			
OM (g kg^-1^)	0.93**	0.80**	-0.46*	0.61**	0.77**	0.72*	0.82***	0.80***	0.86**	-0.91**		
TN (g kg^-1^)	0.90***	0.88*	-0.37*	0.61**	0.80*		0.88*	0.83**	0.89***	-0.88*	0.93***	
C/N			-0.48*						0.35*	-0.49**	0.60**	
AN (mg kg^-1^)	0.91***	0.87*	-0.41*	0.57*	0.77*		0.88**	0.80***	0.91***	-0.89*	0.94*	0.97**

(*p<0.05,

**p<0.01,

***p<0.001).

^†^ETN = extractable total N, AAAs = adsorbed amino acids, UKN = extractable unknown N, EON = extractable organic N, FAAs = free amino acids, HAAs = hydrolyzable amino acids, OM = organic matter, TN = total N, AN = alkali-hydrolyzable N.

## Discussion

The aim of our study was to characterize the concentration and composition of the different forms of N along a mountain elevational gradient. At the Taibai Mountain, temperature and N mineralization rate significantly decreased and organic matter accumulated with the increase of elevation [36]. So the high concentration and proportion of extractable organic N that accounted for soil extractable total N (averaging 67.1%) may have closely related to the high level of plant litter and residue inputs, which are important sources of organic N, especially at high elevations. The accumulation of soil organic matter and the greater soil N content at high elevations are correspondingly beneficial to the increase of soil free, adsorbed and hydrolyzable amino acids, and their positive relationships also confirmed this view (Table 3). The finding was consistent with our first hypothesis that the concentrations of amino acids would increase along the elevational gradient. Our result was somewhat similar to that reported for gradients across successional series. For example, soil free amino acid concentration were greater in late successional coniferous forest (like high elevation forest) than that in early successional deciduous forest (like low elevation forest) [17]. The differences between forest types may vary due to the greater soil protease activity in late successional systems and possibly high elevation systems [42]. Additionally, large amounts of organic compounds are often released by roots and the decomposing microbial biomass under frequent, alternating freezing and thawing [32], which contributes to the increase in amino acid concentrations at the high elevations of Taibai Mountain.

Across the thirteen sampled elevations, the average concentration of soil free amino acids was 1.6 mg kg^-1^ (0.06–5.6 mg kg^-1^), which was similar to the free amino acid concentration reported from boreal forests (0.42–4.9 mg kg^-1^) [17], arctic tundra (1.6–8.3 mg kg^-1^) [43], alpine dry meadows (0.29–2.9 mg kg^-1^) [44], heathland (2.4 mg kg^-1^) [45], and agricultural soils (2.1–7.0 mg kg^-1^) [46]. However, most aforementioned studies have only focused on the free amino acid pool extracted with water or a weak salt solution, which cannot truly reflect soil amino acid pool. For a long time, researchers interpreted that amino acids in soil solution were likely an insignificant source of N for plants, because the concentrations were believed to be too low for appreciable plant uptake and were instead thought to be utilized by microbes [10, 47, 48]. Jones demonstrated that the exchangeable concentration obtained with a strong salt solution was generally several times larger than that of the free amino acids in soil solution [5]. Amino acid adsorption to the soil solid phase not only significantly enhances the potential for amino acid uptake by plants but also lowers amino acid bioavailability to soil microorganisms [26]. In our study, concentration of amino acids extracted with 0.5 mol L^-1^ K_2_SO_4_ was 4 times larger than the free amino acid concentration, their positive relationship indicates that adsorbed amino acids likely represent a potential replenishment source for free amino acids (Table 3, *r* = 0.98, *P*<0.001). Our previous experiment demonstrated that soil adsorbed amino acids provided approximately 15.7–47.3% of plant total N uptake under sterile cultivation [49]. Therefore, further research on plant N uptake in different ecosystems should include the soil adsorbed amino acid pool.

The production of amino acids involves a variety of sources, *e*.*g*., plant litters, root exudates, and microbial cells, whereas its utilization depends on the processes of plant uptake, microbial uptake, and abiotic sorption. Analysis of the amino acid composition of plant litter in boreal forests has revealed high proportions of alanine, glutamine, glutamic acid and histidine [31]. Yu demonstrated that both *Pinus muricata* (*Bishop pine*) and *Cupressus pygmaea* (*pygmy cypress*) leachates in forest ecosystem were dominated by five amino compounds: alanine, aspartic acid, glycine, serine, threonine and glutamic acid [14]. Arginine, asparagine and glutamine are common constituents of plant xylem and phloem [24, 50], and microorganisms, whose cell walls contain high proportions of alanine, aspartic acid, and glutamic acid, are also likely sources of soil amino acids [14, 51]. The pool of hydrolyzable amino acids was dominated by alanine (6.8%), aspartic acid (5.7%), glutamic acid (5.7%), glycine (10.6%), histidine (29.6%), serine (5.2%) and threonine (5.3%) across the elevational gradient, which is partly in accordance with the results of the aforementioned studies. Notable among our results was the larger proportion of histidine relative to other amino acids in soil. There may be two reasons to explain this result: 1) basic amino acids possess a strong positive charge, especially in low pH soils [17, 52]; and 2) as organic matter accumulates with elevational gradient, there is an associated increase in cation exchange capacity [53], so more amino acids (especially histidine) may be adsorbed onto the soil.

Between elevations, there was large variation in concentrations of the different individual amino acids (Fig 2), yet results of multiple simple linear regressions demonstrated that the major amino acid composition (76.3% of total, including alanine, arginine, aspartic acid, glycine, histidine, leucine, phenylalanine, serine, threonine and valine) showed no significant correlation with elevation (*p*>0.10, Fig 4), which is in contrast to our second hypothesis that amino acids with simple molecular structure would be prevalent at low elevations, and amino acids with complex aromatic structure and high molecular weight would be prevalent at high elevations. A review of other studies suggest that there are common soil amino acids across a variety of natural and managed ecosystems from different climates [17]. This suggests that soil amino acids likely originate from similar ecosystem components or through similar biochemical processes. In contrast, some authors reported that the prevalence of alanine, aspartic acid, glutamic acid and glycine is likely due to their relative resistance to microbial decomposition [54]. A small proportion (19.2%) of amino acids had a marginally significant or significant association with elevation, such as glutamic acid, isoleucine, methionine, proline and tyrosine, which is consistent with reviews in others studies across a variety of different soils [19, 52]. For both plants and soil microbes, soil N compositions depend on both the organism physiological capacity to take up soil inorganic N and amino acids, and soil N bioavailability. The presence of significant relationships between amino acid proportions and elevations may be attributed to the imbalance between amino acid uptake rate and N mineralization rate with the increase in elevation. The production and utilization of amino acids within plant or microbial cells, as well as the ecosystem processes are all significantly related to soil amino acid composition. Therefore, elucidating which ecosystem components or processes regulate the relative abundance of individual amino acid requires further investigation.

According to their functional classification, neutral amino acids predominated and accounted for 47.8% of soil total hydrolyzable amino acids. A possible explanation for this finding is that soil microbial activity causes a turnover of protein material and selectively preserves the neutral compounds due to the interaction with other soil components. Acidic amino acids only accounted for a small proportion of soil total hydrolyzable amino acids, which contrasted with the composition of free amino acids that were dominated by aspartic acid and/or glutamic acid in other studies [17, 28]. This discrepancy may be due to ecosystem differences but is more likely a reflection of the differences between free amino acids and hydrolyzable amino acids. The pool of soil free amino acids was dominated by neutral and acidic amino acids that were easily extracted by water or weak salt solution, whereas in our study, the hydrolyzable pool likely included a large proportion of complex protein N that was affected by soil type, litter input and the environment.

Plants can directly take up both the inorganic N and organic N. Soil amino acids, as the main existing form of organic N, constitute a high proportion of plant N uptake in many terrestrial ecosystems [9–12]. Because N composition and N availability commonly regulate plant biomass production as well as species diversity in terrestrial ecosystems [55, 56], the high proportion of amino acid-N at high elevation of Taibai Mountain has important ramifications for our understanding of ecological processes and how plant N uptake vary by temperature and species. However, little is known about the relationship between hydrolyzable amino acids content and soil total N content along the elevational gradient of Taibai Mountain. Investigating this problem requires us to fully understand the direct bioavailability of soil organic N to plants.

## Conclusions

In conclusion, this study demonstrated strong elevational variations in the concentrations of soil extractable total N, extractable organic N and amino acid N due to the increase of soil organic matter and the greater N content along the elevational gradient of Taibai Mountain. Concentration of soil extractable organic N was significantly greater than the concentration of soil extractable inorganic N (including NO_3_^−^-N and NH_4_^+^-N). The seven dominant amino acids were histidine, alanine, glutamic acid, glycine, aspartic acid, threonine and serine across the elevational gradient. Irrespective of the significant variations in soil properties along the elevational gradient, the abundance of most major amino acid (about 76.3% of total, including alanine, arginine, aspartic acid, glycine, histidine, leucine, phenylalanine, serine, threonine and valine) did not vary appreciably with elevation (*p*>0.10), which suggests that many major soil amino acids likely originate from a common source or through similar biochemical processes. However, the proportions of isoleucine, methionine and tyrosine varied with elevation, and may indicate an imbalance between amino acid uptake rate and N mineralization rate with the increase of elevation.

On average, most soil amino acids (5 times) were bound to soil particles and a much smaller proportion were free in soil solution. This suggests that soil adsorbed amino acids may help to replenish the free amino acid in the soil solution. Therefore, further research on plant N uptake in different ecosystems should incorporate the uptake of soil adsorbed amino acids.
